# A Simplified Physiology–Laboratory Score (PLS4) for Early Risk Stratification in Acute Cholangitis

**DOI:** 10.3390/jcm15207842

**Published:** 2026-10-11

**Authors:** Jaewon Lee, Moon Suk Choi, Yujin Lee, Jeseop Lee, Jungnam Lee, Tae-Youn Kim

**Affiliations:** 1 Department of Emergency Medicine, College of Medicine, Inha University Hospital, Inha University, 27, Inhang-ro, Jemulpo-gu, Incheon 22332, Republic of Korea; abcdg96@naver.com (J.L.); eyeblack99@gmail.com (Y.L.); swf365@naver.com (J.L.); 2 Department of Surgery, College of Medicine, Inha University Hospital, Inha University, Incheon 22332, Republic of Korea; insilion@gmail.com; 3 Department of Internal Medicine, College of Medicine, Inha University Hospital, Inha University, Incheon 22332, Republic of Korea; jungnamlee801105@gmail.com

**Keywords:** cholangitis, emergency service, hospital, intensive care units, risk assessment, severity of illness index, prognosis

## Abstract

**Objectives:** Acute cholangitis is a common biliary infection in the emergency department (ED) that can progress rapidly to septic shock and multiorgan dysfunction. The Tokyo Guidelines 2018 (TG2018) is the current reference standard for severity assessment but may be cumbersome during the initial ED encounter. We aimed to develop a simplified physiology–laboratory score (PLS4) computable from four routine bedside variables and to compare its early risk-stratification performance with TG2018. **Materials and Methods:** This single-center retrospective observational cohort study included 482 adult patients with acute cholangitis (International Classification of Diseases, 10th Revision code K83.0) presenting to a tertiary university hospital ED between January 2016 and December 2021. Primary outcomes were ICU admission, vasopressor use, and prolonged hospital stay (length of stay ≥ 14 days); intubation was analyzed as an exploratory outcome. PLS4 assigned 1 point each for albumin < 3.5 g/dL, systolic blood pressure ≤ 100 mmHg, heart rate ≥ 100/min, and prothrombin time–international normalized ratio ≥ 1.2 (range 0–4). Discrimination was assessed by receiver operating characteristic curves, with bootstrap 95% confidence intervals and bootstrap optimism-corrected internal validation. **Results:** ICU admission occurred in 81 patients (16.8%), vasopressor use in 53 (11.0%), and prolonged stay in 111 (23.0%). The areas under the curve (AUCs) for ICU admission were 0.850 for TG2018 and 0.800 for PLS4 (*p* = 0.116), and the optimism-corrected AUC of PLS4 was 0.779. For vasopressor use, which is part of the TG2018 grade III definition, the AUCs were 0.936 and 0.843. When PLS4 was added to TG2018, the AUC for ICU admission increased to 0.886. At PLS4 ≥ 3, specificity reached 0.978 for ICU admission and 0.970 for vasopressor use, with event rates rising stepwise from 5.96% to 100% and 2.55% to 90.0%, respectively. **Conclusions:** In this single-center derivation study, PLS4 showed high specificity and added prognostic information to TG2018. It should be validated in other centers before clinical use.

## 1. Introduction

Acute cholangitis is a common biliary infection encountered in the emergency department (ED) among patients presenting with abdominal pain, fever, and jaundice. Prompt diagnosis and management are essential to prevent rapid deterioration [1]. Delayed antimicrobial therapy and biliary drainage can lead to septic shock, multiorgan dysfunction, intensive care unit (ICU) admission, and death [2]. Despite advances in broad-spectrum antimicrobial therapy and biliary decompression, recent reviews have reported mortality rates of up to 15% [3]. Among critically ill patients admitted to the ICU, in-hospital mortality reaches approximately 28–29% [4]. Although Charcot’s triad remains a classic diagnostic concept, its sensitivity in clinical practice is limited [5]. The original Tokyo Guidelines, introduced in 2007, established the first standardized diagnostic criteria and severity assessment framework for acute cholangitis [6]. The 2018 update (TG2018) refined the diagnostic criteria and severity grading and has been validated as the worldwide standard [7]. TG2018 also established management bundles linking severity grade to treatment, including the timing of biliary drainage [8].

Validation studies have consistently demonstrated that the Tokyo Guidelines outperform classical clinical signs such as Charcot’s triad for diagnosing acute cholangitis [9]. Studies in patients with choledocholithiasis have likewise reported high diagnostic sensitivity and specificity [10]. TG18 also improves diagnostic specificity by reducing reader-dependent variability in severity grading [11]. Comparisons of TG07 and TG13 found similar diagnostic rates (approximately 85%) but differences in severity classification and identified hypoalbuminemia (≤25 g/L) as an independent predictor of 30-day mortality [12]. External validation in an Indonesian cohort reported 84% sensitivity and 96% specificity for TG18, with grade III independently predicting mortality [13]. Recent studies have also demonstrated a mortality benefit from urgent endoscopic retrograde cholangiopancreatography (ERCP) guided by TG2018 severity across age groups [14].

Despite its clinical utility, TG2018 can be difficult to apply during the initial ED evaluation, when decisions often rely only on vital signs and basic laboratory results [1]. In an ED study, TG2018 predicted ICU admission with an area under the curve (AUC) of 0.850, supporting its prognostic value while highlighting the need for simpler bedside tools [15]. Several biomarkers and prognostic indices have been proposed, including presepsin [16]. Procalcitonin has outperformed white blood cell count and C-reactive protein for predicting severe acute cholangitis (AUC 0.90) [17]. The red cell distribution width-to-albumin ratio has been proposed as a prognostic marker for in-hospital mortality and ICU admission in patients requiring biliary drainage [18]. A combined neutrophil-to-lymphocyte ratio and prognostic nutritional index score achieved an AUC of approximately 0.85 for severity prediction [19]. The neutrophil percentage-to-albumin ratio has also been associated with 30-day mortality in patients with septic cholangitis [20]. However, these markers often require additional laboratory tests or complex calculations, limiting routine bedside use during initial ED triage.

The timing of biliary drainage is another key prognostic factor in acute cholangitis [21]. Among critically ill patients, drainage within 24 h has been associated with less persistent organ failure and shorter hospital stay [22]. A population-based analysis showed that ERCP should not be delayed beyond 48 h in patients with acute cholangitis due to choledocholithiasis [23]. For acute cholangitis caused by distal malignant biliary obstruction, urgent ERCP within 24 h has been associated with lower 30-day and 180-day mortality [24]. A national analysis further found that ERCP performed >72 h after admission was associated with increased in-hospital mortality [25]. Likewise, in a multicenter ICU cohort, biliary decompression delayed >48 h after ICU admission independently predicted in-hospital mortality [4].

Previous studies have identified multiple clinical and laboratory predictors of adverse outcomes in acute cholangitis [26]. In TG07-managed cohorts, serum albumin ≤ 2.8 g/dL and prothrombin time–international normalized ratio (PT-INR) > 1.5 independently predicted mortality and refractory disease [27]. Comparisons of TG07 and TG13 also identified hypoalbuminemia and intrahepatic obstruction as independent predictors of 30-day mortality [12]. In an ICU cohort, anticoagulant use, catecholamine support, mechanical ventilation, and delayed antibiotic therapy were independently associated with mortality in severe acute cholangitis [28]. Collectively, these findings suggest that a limited number of bedside physiologic and laboratory variables, including albumin and PT-INR, provide meaningful prognostic information during the initial evaluation [27].

More complex prognostic models have also been developed. The Mortality Risk for Acute Cholangitis (MAC) score was derived from nearly 1000 episodes to predict in-hospital mortality [29]. A simpler prognostic score has since been proposed for early prediction of 30-day mortality [30]. Machine learning models, including XGBoost-based pipelines, have also been externally validated for predicting outcomes in cholelithiasis-induced acute cholangitis [31]. However, these models require multiple variables and computational steps, limiting their practicality during the initial bedside assessment [31].

In this single-center cohort, we aimed to develop a simplified physiology–laboratory score (PLS4), calculated from four routinely available bedside variables, and compare its early risk stratification performance with the TG2018 severity grading system [7]. Our objective was not to replace TG2018 as the standard severity classification but to determine whether a simple adjunct score using a minimal number of variables could improve early ED risk stratification [6]. Because the score was developed and tested in the same cohort, this study should be regarded as a derivation study, and external validation is needed before clinical use.

## 2. Methods

### 2.1. Study Design and Population

We screened consecutive adult patients (≥19 years) admitted with acute cholangitis between January 2016 and December 2021. This retrospective observational study was conducted in the ED of a tertiary university hospital that treats approximately 35,000 patients annually, including about 100 patients with acute cholangitis. The study was approved by the Institutional Review Board (IRB No. DUIH 2021-10-016-002) and complied with the ethical principles of the 1975 Declaration of Helsinki.

Because of the retrospective observational design, informed consent was waived. Patient records were anonymized before analysis. Electronic medical records were reviewed, and patients discharged with the International Classification of Diseases, 10th Revision codes for acute cholangitis (K83.0) or choledocholithiasis with cholangitis (K80.3x) were screened.

We screened 502 consecutive patients identified by discharge codes for acute cholangitis (K83.0) and choledocholithiasis with cholangitis (K80.3x) between January 2016 and December 2021. After excluding 20 patients without serum laboratory tests, 482 patients were included in the analysis (Figure S1). Data collected included age, comorbidities, presentation vital signs, laboratory findings, biliary drainage, ICU admission, vasopressor use, intubation, and hospital length of stay. The mean age was 71.6 ± 15.2 years (median, 75 years; interquartile range, 63–83). Study design and reporting followed established transparency standards for prediction model research, and risk of bias was assessed according to current methodological recommendations [32].

All included patients were diagnosed with acute cholangitis based on the TG2018 diagnostic criteria, including imaging findings of biliary dilatation or an underlying cause. The discharge codes were used only to identify eligible patients.

### 2.2. Outcomes

Because only eight patients (1.7%) died, mortality was excluded from the primary outcomes for score development. Primary outcomes were ICU admission, vasopressor use, and prolonged hospital stay (length of stay ≥ 14 days). Intubation was evaluated as an exploratory outcome because of the low event rate. Among 482 patients, ICU admission occurred in 81 (16.8%), vasopressor use in 53 (11.0%), prolonged hospital stay in 111 (23.0%), and intubation in 14 (2.9%).

Vasopressor use was defined as intravenous norepinephrine, epinephrine, dopamine, or vasopressin administered for hemodynamic support at any dose or duration in the ED. ICU admission was defined as admission to a medical or surgical intensive care unit. Our hospital does not have a separate step-down unit, and observation in the ED was not counted as ICU admission. Intubation was defined as endotracheal intubation with invasive mechanical ventilation of any duration. Prolonged stay was defined as a hospital length of stay ≥ 14 days.

The TG2018 severity grade was determined in the emergency department (ED) during initial management, based on vital signs at presentation, initial laboratory results, and initial mental status. Because grading was performed during ED treatment, vasopressor use in the ED was counted as meeting the cardiovascular criterion of grade III.

### 2.3. Candidate Variables and PLS4 Construction

Candidate variables were selected from vital signs and routine laboratory tests obtained during the initial ED evaluation. All score variables were taken from the first measurements at ED presentation, before fluid resuscitation or vasopressor administration. Univariable analyses for ICU admission and vasopressor use showed that albumin, systolic blood pressure (SBP), PT-INR, blood urea nitrogen (BUN), and heart rate (HR) consistently had higher AUCs, consistent with prognostic factors reported in TG-graded cohorts [27]. Albumin, SBP, HR, and PT-INR were selected because they are routinely available and have well-established physiologic relevance [15]. The final PLS4 included four binary variables: albumin < 3.5 g/dL, SBP ≤ 100 mmHg, HR ≥ 100/min, and PT-INR ≥ 1.2. Each variable was assigned 1 point, producing a total score of 0–4. Cut-offs were selected to balance discrimination with clinical simplicity. With 81 ICU admissions and five candidate variables, the number of events per candidate variable was approximately 16. Rather than developing a regression coefficient-weighted model, we intentionally created a simple additive score for bedside ED use.

### 2.4. Comparator and Statistical Analysis

TG2018 severity grading served as the comparator. Predictive performance of individual variables and both scoring systems was evaluated using receiver operating characteristic (ROC) curves and the AUC. Sensitivity, specificity, positive predictive value, negative predictive value, and event rates by score category were also evaluated. Internal validation was performed using bootstrap optimism correction with 1000 resamples. Because the candidate variables and cut-offs were chosen using the study data, the whole derivation process was repeated in each bootstrap sample. This included selecting four of the five candidate variables (albumin, systolic blood pressure, heart rate, PT-INR, and blood urea nitrogen) based on the mean univariable AUC for ICU admission and vasopressor use, choosing each cut-off by the Youden index for ICU admission, and building the equally weighted score. Optimism was calculated as the mean difference between the AUC of the re-derived score in the bootstrap sample and its AUC in the original sample, and this value was subtracted from the apparent AUC. TG2018 has no fitted parameters, so no optimism correction was applied to it. Reporting followed the TRIPOD statement. Statistical analyses were performed using IBM SPSS Statistics (version 23.0; IBM, Armonk, NY, USA), MedCalc 17.5.3 (MedCalc Software, Ostend, Belgium), R 4.2.0, and Python 3.13.0 in Google Colab.

Confidence intervals for AUCs were obtained from 2000 bootstrap resamples stratified by outcome. Differences in AUC between PLS4 and TG2018 were tested using paired bootstrap resampling. All tests were two-sided, and *p* < 0.05 was considered statistically significant.

Calibration was assessed in three ways: observed event proportions across score categories, the bootstrap optimism-corrected calibration slope and calibration-in-the-large of a logistic model with the score as the only predictor, and a Hosmer–Lemeshow-type test with score categories as groups.

To assess incremental value, a logistic model with TG2018 grade was compared with a model including both TG2018 grade and PLS4, using the change in AUC and the category-free net reclassification improvement (NRI). Clinical usefulness was evaluated by decision-curve analysis for ICU admission and vasopressor use. To estimate what is lost by equal weighting, PLS4 was compared with a coefficient-weighted logistic model using the same four predictors and with a five-variable model including BUN. In-hospital mortality was reported descriptively by PLS4 category and TG2018 grade.

## 3. Results

### 3.1. Cohort Characteristics and Event Rates

Among 482 patients, ICU admission occurred in 81 (16.8%), vasopressor use in 53 (11.0%), prolonged hospital stay (length of stay ≥ 14 days) in 111 (23.0%), and intubation in 14 (2.9%). ERCP was performed in 324 patients (67.2%) and percutaneous transhepatic biliary drainage in 49 (10.2%). The mean hospital stay was 11.1 days. ICU admission and vasopressor use had sufficient event counts for model development, whereas intubation was evaluated only as an exploratory outcome (Table 1).

### 3.2. Univariable Predictive Performance

For ICU admission, TG2018 achieved the highest AUC (0.850). Among individual physiologic and laboratory variables, albumin had an AUC of 0.771, followed by PT-INR (0.754), SBP (0.737), and BUN (0.713). For vasopressor use, TG2018 again had the highest AUC (0.936), followed by albumin (0.820), SBP (0.807), PT-INR (0.796), BUN (0.771), and HR (0.712). These findings support the association of hypoalbuminemia, hypotension, tachycardia, and coagulopathy with early clinical deterioration in acute cholangitis (Table 2).

### 3.3. PLS4 Score Distribution

PLS4 included albumin < 3.5 g/dL, SBP ≤ 100 mmHg, HR ≥ 100/min, and PT-INR ≥ 1.2. Scores were 0 in 235 patients, 1 in 141, 2 in 63, 3 in 33, and 4 in 10. ICU admission and vasopressor use increased progressively with higher PLS4 scores (Table 3). The intubation rate did not increase consistently with the score (21.2% at score 3 vs 10.0% at score 4), probably because of the small number of patients in these categories (n = 33 and n = 10). Intubation results are therefore presented as exploratory, with Wilson score 95% confidence intervals (Table 3).

### 3.4. PLS4 Versus TG2018 Performance

**Figure 1:**
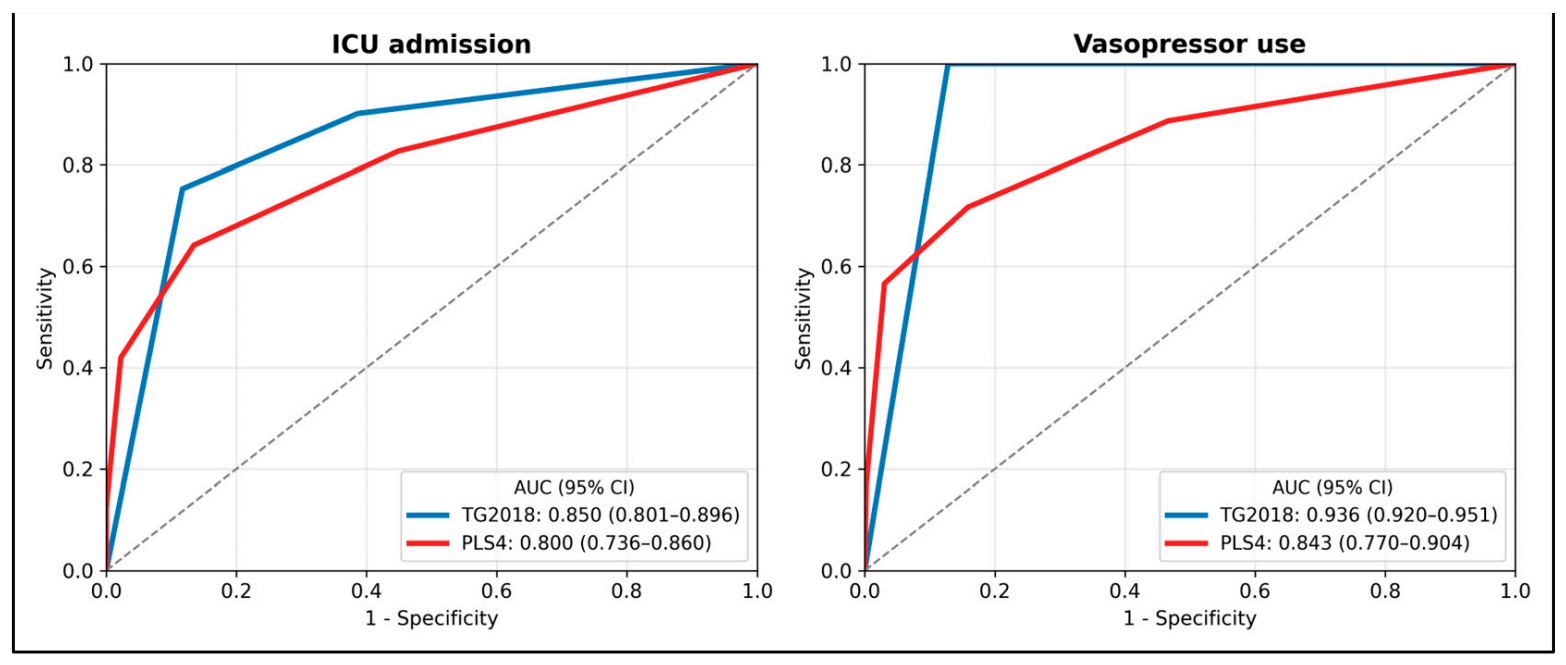
Receiver operating characteristic (ROC) curves comparing the predictive performance of TG2018 and the simplified physiology–laboratory score (PLS4) for ICU admission and vasopressor use in patients with acute cholangitis. AUCs (95% confidence intervals) were 0.850 (0.801–0.896) versus 0.800 (0.736–0.860) for ICU admission and 0.936 (0.920–0.951) versus 0.843 (0.770–0.904) for vasopressor use.

The AUCs of TG2018 were higher than those of PLS4 for all outcomes, but the difference was statistically significant only for vasopressor use (ΔAUC 0.093, *p* = 0.001). For ICU admission, the primary comparison, there was no significant difference (PLS4 0.800 [95% CI 0.736–0.860] vs TG2018 0.850 [0.801–0.896]; ΔAUC 0.051, *p* = 0.116). After correction for optimism from variable and cut-off selection, the AUC of PLS4 was 0.779 for ICU admission (optimism 0.021), 0.828 for vasopressor use (0.015), 0.664 for prolonged stay (0.005), and 0.756 for intubation (0.011) (Table 4) (Figure 1).

TG2018 grade III includes cardiovascular dysfunction defined by the need for catecholamines, so the comparison for vasopressor use is partly circular. All 53 patients who received vasopressors were grade III (49.1% of the 108 grade III patients), and none of the 374 patients with grade I or II received vasopressors. The AUC of 0.936 for TG2018 therefore mainly reflects its own grade III definition, and the difference between PLS4 and TG2018 (0.843 vs. 0.936) should be regarded as the upper limit of the true difference. ICU admission is not part of the severity grading, so it was chosen as the primary comparison. For this outcome, there was no significant difference between PLS4 and TG2018 (ΔAUC 0.051, *p* = 0.116).

### 3.5. Outcome Rates by PLS4 Category

**Figure 2:**
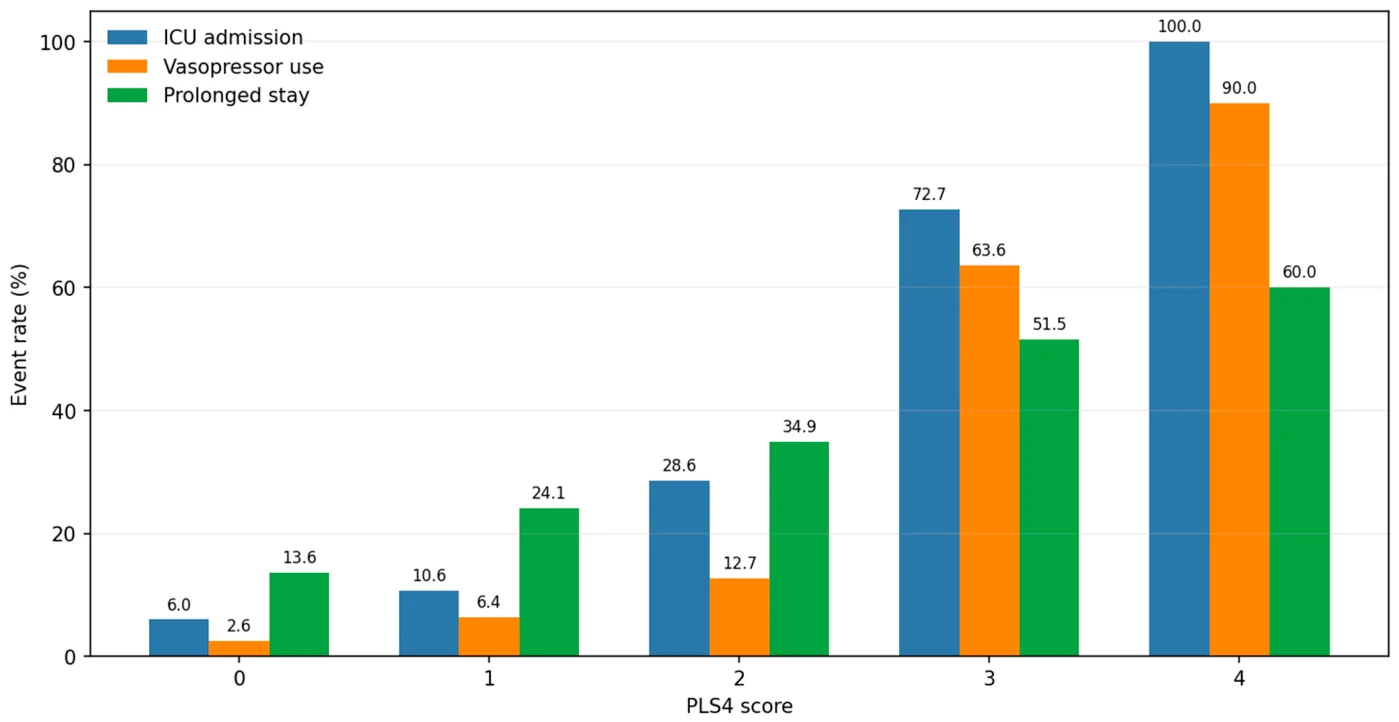
Stepwise increase in adverse clinical outcomes according to PLS4 score in acute cholangitis. ICU admission increased from 5.96% at score 0 to 100% at score 4, whereas vasopressor use increased from 2.55% to 90.0%. Prolonged hospital stay also increased across score categories.

ICU admission rates by PLS4 score were 5.96% for score 0, 10.64% for 1, 28.57% for 2, 72.73% for 3, and 100% for 4. Corresponding vasopressor use rates were 2.55%, 6.38%, 12.70%, 63.64%, and 90.0%. Prolonged hospital stay increased more gradually, from 13.62% at score 0 to 60.0% at score 4. Intubation rates varied across categories because of the low event counts. Overall, PLS4 showed a clear stepwise increase in risk, particularly for ICU admission and vasopressor use (Figure 2).

Eight patients (1.7%) died in hospital. By PLS4 score, crude mortality was 0/235 (0%), 0/141 (0%), 2/63 (3.2%), 5/32 (15.6%), and 1/10 (10.0%) for scores 0 to 4. By TG2018 grade, it was 0/254 (0%), 1/120 (0.8%), and 7/107 (6.5%) for grades I to III. Because the number of deaths was small, no statistical comparison was made.

### 3.6. Operating Characteristics at Clinically Relevant Cut-Offs

**Figure 3:**
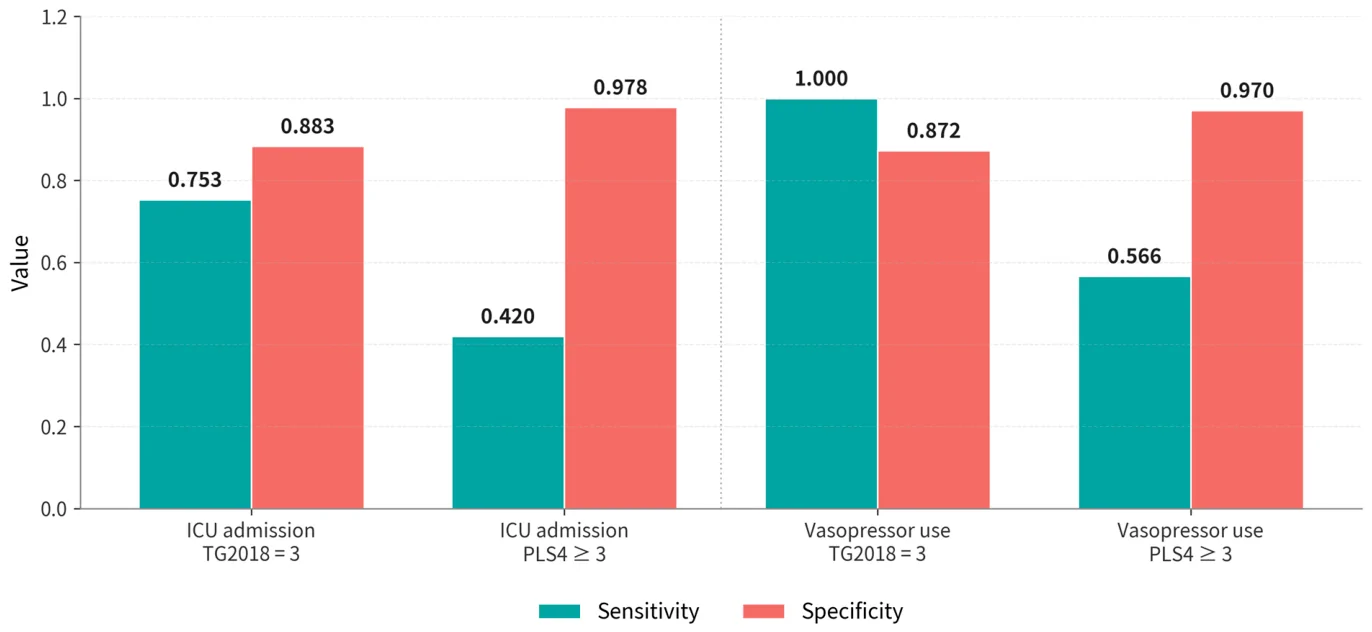
Operating characteristics of TG2018 grade 3 and PLS4 ≥ 3 for ICU admission and vasopressor use. For ICU admission, TG2018 grade 3 had a sensitivity of 0.753 and specificity of 0.883, whereas PLS4 ≥ 3 had a sensitivity of 0.420 and specificity of 0.978. For vasopressor use, TG2018 grade 3 had a sensitivity of 1.000 and specificity of 0.872, whereas PLS4 ≥ 3 had a sensitivity of 0.566 and specificity of 0.970.

For ICU admission, TG2018 ≥ 2 yielded a sensitivity of 0.901 and specificity of 0.613, whereas TG2018 grade 3 yielded a sensitivity of 0.753 and specificity of 0.883. PLS4 ≥ 2 yielded a sensitivity of 0.642 and specificity of 0.865, whereas PLS4 ≥ 3 yielded a sensitivity of 0.420 and specificity of 0.978. For vasopressor use, TG2018 grade 3 achieved a sensitivity of 1.000 and specificity of 0.872, whereas PLS4 ≥ 3 achieved a sensitivity of 0.566 and specificity of 0.970 (Table 5) (Figure 3).

### 3.7. Incremental Value over TG2018 and Calibration

**Figure 4:**
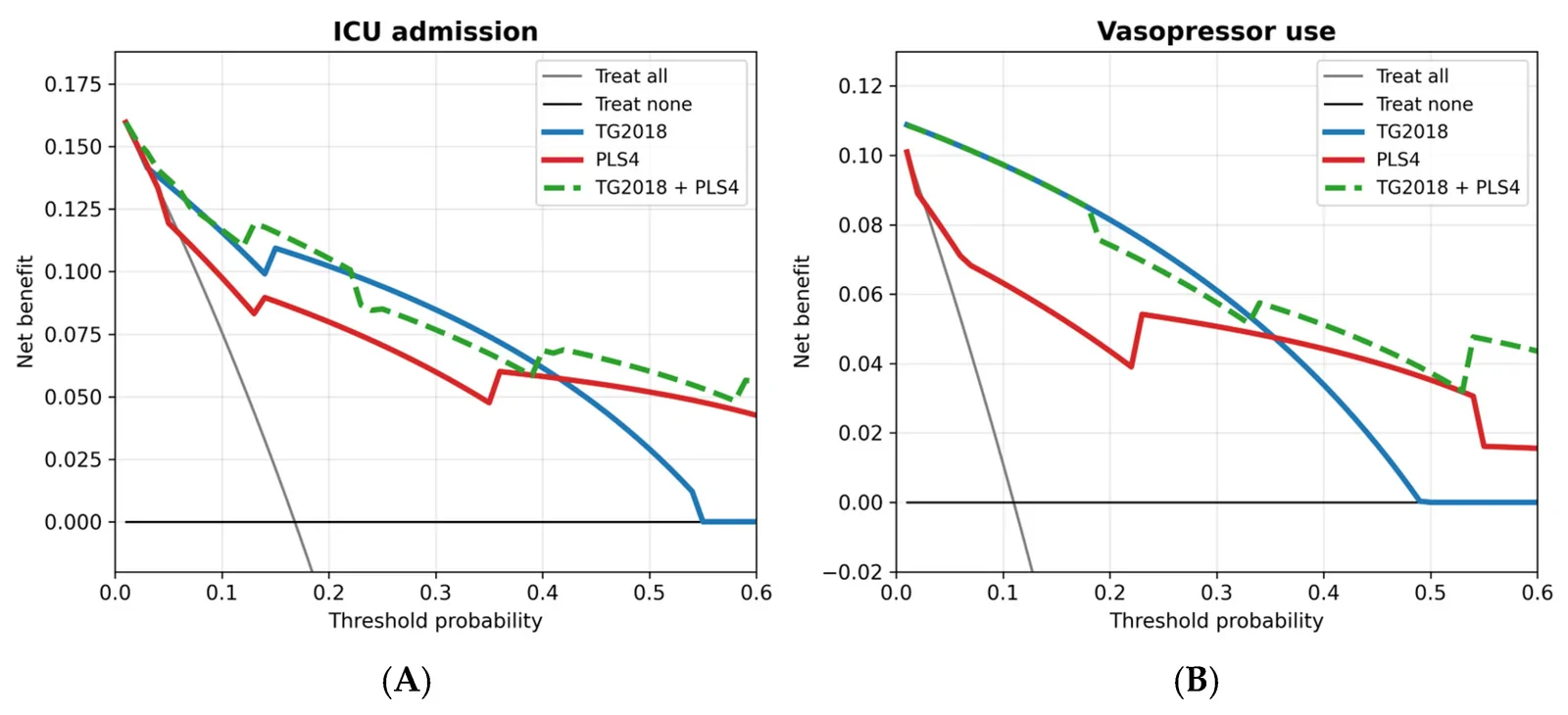
Decision curve analysis of TG2018, PLS4, and the combined model (TG2018 + PLS4). (**A**) ICU admission and (**B**) vasopressor use, showing the net benefit of TG2018, PLS4, and the combined model (TG2018 + PLS4) compared with treating all or no patients.

When PLS4 was added to TG2018, the AUC increased from 0.850 to 0.886 for ICU admission (ΔAUC 0.036, *p* < 0.001) and from 0.936 to 0.966 for vasopressor use (ΔAUC 0.030, *p* < 0.001). The category-free net reclassification improvement was +0.844 for ICU admission and +0.875 for vasopressor use. In the decision-curve analysis (Figure 4), TG2018 had a greater net benefit than PLS4 at low threshold probabilities, whereas PLS4 had a greater net benefit at thresholds above about 35–40%, which fits its intended use for identifying high-risk patients. The combined model showed a net benefit similar to or greater than that of TG2018 alone at most thresholds.

**Figure 5:**
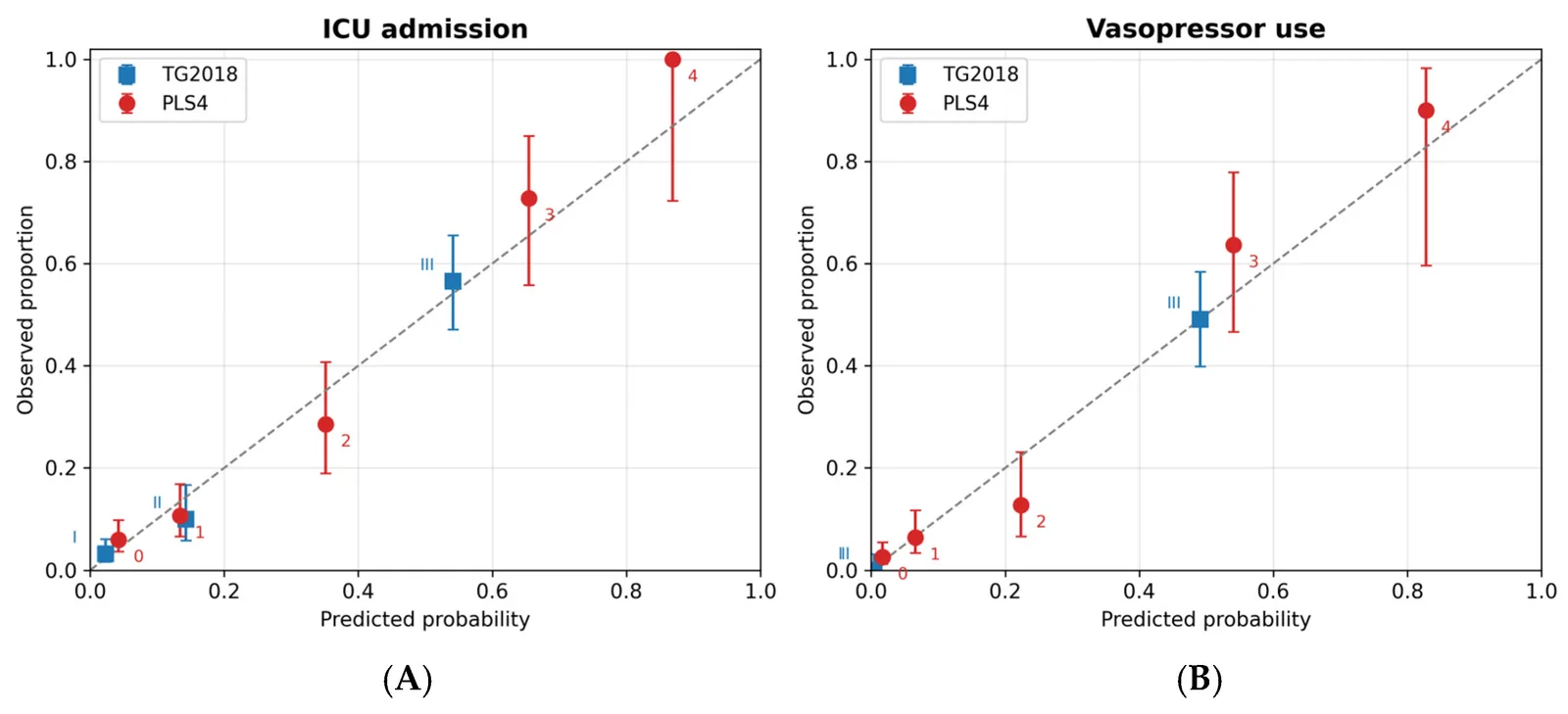
Calibration of TG2018 grade and PLS4 score. (**A**) ICU admission and (**B**) vasopressor use. Observed event proportions with 95% confidence intervals are plotted against predicted probabilities from score-only logistic models; labels indicate the PLS4 score (0–4) or TG2018 grade (I–III), and the dashed line indicates perfect calibration.

Observed event rates increased almost steadily across PLS4 scores and TG2018 grades for both primary outcomes (Figure 5). After bootstrap optimism correction, the calibration slope of PLS4 was 0.93 for ICU admission and 0.95 for vasopressor use, and calibration-in-the-large was 0.02 and 0.03, respectively. The Hosmer–Lemeshow-type test showed no evidence of poor fit (*p* = 0.103 and *p* = 0.108).

### 3.8. Equal Versus Coefficient-Weighted Scoring

A logistic model using the same four predictors with estimated coefficients had AUCs of 0.808 for ICU admission and 0.889 for vasopressor use, compared with 0.800 and 0.843 for PLS4 with equal weights. Thus, little discrimination for the primary outcome was lost by using a simple score (ΔAUC 0.008). Adding BUN as a fifth variable gave AUCs of 0.820 and 0.891 (Supplementary Table S1). BUN was not included in PLS4 to keep the score simple, because it added little and was correlated with age and renal function.

### 3.9. Biliary Decompression by TG2018 Severity Grade

According to TG2018 severity, 254 patients (52.7%) were grade I, 120 (24.9%) grade II, and 108 (22.4%) grade III (Table 1). The median time to ERCP was 29.4 h, 28.5 h, and 50.1 h for grades I, II, and III, and ERCP was performed within 24 h in 37.5%, 35.7%, and 25.0%, respectively. The median time to PTBD was 38.4 h, 25.4 h, and 21.3 h (Table 6).

## 4. Discussion

In this single-center cohort of 482 patients with acute cholangitis, we developed a four-variable physiology–laboratory score (PLS4) and compared it directly with TG2018. TG2018 showed higher AUCs, but the difference was statistically significant only for vasopressor use, which is part of the TG2018 grade III definition [7]. For ICU admission, the primary comparison, discrimination did not differ significantly. Nevertheless, PLS4 provided clinically meaningful discrimination for ICU admission and vasopressor use, with event rates increasing markedly as the score increased. Adding PLS4 to TG2018 improved discrimination for ICU admission (AUC from 0.850 to 0.886), suggesting that the two provide complementary information. These findings suggest that PLS4 is not a replacement for TG2018 but a simple, low-complexity adjunct for early ED triage [15].

The numerically higher discrimination of TG2018 is expected given its broader design [7]. Beyond routine vital signs and laboratory values, TG2018 incorporates multiple indicators of organ failure and disease severity [6]. Previous validation studies have consistently supported its diagnostic and prognostic performance across diverse settings [11]. External validation has also confirmed TG18 grade III as an independent predictor of in-hospital mortality [13]. In addition, TG2018-guided urgent ERCP has been associated with lower mortality across age groups [14]. Our findings are consistent with these reports and reinforce TG2018 as the reference standard for severity assessment [15].

The primary strength of PLS4 is its simplicity because all four variables are routinely available during the initial ED evaluation [15]. Hypoalbuminemia reflects systemic inflammation, nutritional status, and impaired hepatic synthetic function and has been associated with mortality and refractory cholangitis in TG-graded cohorts [27]. Other cohort studies have also identified hypoalbuminemia as an independent predictor of 30-day mortality in acute cholangitis [12]. Hypotension and tachycardia reflect circulatory instability and septic stress and have been identified as early prognostic markers in multivariable analyses [26]. Elevated PT-INR reflects coagulopathy and hepatic dysfunction and has been independently associated with disease severity and adverse outcomes in TG-graded cohorts [27].

PLS4 demonstrated high specificity at higher score thresholds. A PLS4 score ≥ 3 achieved a specificity of 0.978 for ICU admission and 0.970 for vasopressor use, suggesting that it may help identify patients at substantially increased risk in the ED [15]. Rather than serving as a broad screening tool, PLS4 may be better suited as a rule-in tool for rapidly identifying patients who require closer monitoring or escalation of care.

In our elderly cohort (median age 75 years), age ≥ 75 years alone meets one of the grade II criteria. This may reduce the ability of TG2018 to separate patients at the lower end of severity and may partly explain its low positive predictive value for ICU admission at grade II or higher (0.320). The results may be different in younger patients, and this should be examined in external validation. For prolonged hospital stay, the AUC difference between TG2018 and PLS4 was small. Hospital stay is influenced not only by initial physiologic status but also by disease etiology, drainage feasibility, procedural delays, and comorbidities. A population-based study showed that ERCP delayed > 48 h was associated with longer hospital stay and higher in-hospital mortality [23]. Therefore, PLS4 may be more useful for predicting early clinical deterioration than prolonged hospital stay. Patients with the most severe disease underwent ERCP later, probably because hemodynamic stabilization was needed before endoscopy, whereas PTBD, when performed, was earliest in grade III.

This interpretation is consistent with ICU-based studies of acute cholangitis. A multicenter ICU study identified SOFA score, lactate, total bilirubin, obstruction etiology, and biliary decompression delayed >48 h as independent predictors of in-hospital mortality [4]. Another study linked catecholamine use, mechanical ventilation, and delayed antibiotic therapy to poor outcomes [28]. Thus, an effective early triage tool should identify clinically significant physiologic derangement while recognizing patients most likely to require treatment escalation [1].

From a methodological standpoint, PLS4 was intentionally designed using a minimal variable set [33]. Internal validation was performed using bootstrap optimism correction with variable and cut-off selection repeated in each resample, and the estimated optimism was small (0.021 for ICU admission and 0.015 for vasopressor use) [32]. This approach also aligns with current recommendations for transparent reporting of prediction models using regression or machine learning methods [34]. This observational study was reported according to the STROBE guidelines [35].

Recent prognostic research in acute cholangitis has expanded beyond simple scoring systems. The MAC score was developed to predict in-hospital mortality [29], and additional simplified scores have been proposed for early prediction of 30-day mortality [30]. Machine learning models, including XGBoost, have also demonstrated promising performance in multicenter retrospective cohorts of cholelithiasis-induced acute cholangitis [31]. However, external validation of similar machine learning models has often yielded inconsistent results across clinical settings [36]. Therefore, independent external validation remains essential before widespread clinical implementation of any prognostic model, including PLS4 [37].

### Limitations

This study has several limitations. First, this was a retrospective single-center study, so selection and information bias cannot be excluded [35]. Second, intubation was evaluated only as an exploratory outcome, and mortality was excluded from the primary outcomes because of the low event count. Third, although equally weighted binary variables improve clinical applicability, they reduce information contained in continuous variables [33]. We tried to re-analyze only patients classified as grade III before any vasopressor therapy, but this was not possible. The PaO_2_/FiO_2_ ratio and the timing of vasopressor initiation relative to grading in the ED were not available in our dataset, and hypotension at presentation (SBP < 90 mmHg) identified only 19% of grade III patients, so re-grading based on presentation data alone would have produced a substantially different classification. Calendar dates were not kept in the extracted dataset, so analyses by time period, including the COVID-19 period, could not be performed. The etiology of cholangitis (stones, malignancy, or benign stricture) was not systematically recorded and could not be obtained for the whole cohort. Serum albumin has a half-life of about three weeks and reflects chronic nutritional and liver status rather than acute illness; this is a limitation of the score, but it may also contribute to its prognostic value. Finally, although internal validation was performed using bootstrap optimism correction [32], external validation in an independent cohort was not performed [37]. Because external validation of similar prediction models remains limited [36], PLS4 should be validated in independent cohorts before widespread clinical implementation.

## 5. Conclusions

PLS4, a simple physiology–laboratory score based on four bedside variables (albumin, SBP, HR, and PT-INR), provided meaningful early risk stratification for ICU admission and vasopressor use in patients with acute cholangitis. Although TG2018 should remain the reference standard for severity assessment, PLS4 may serve as a practical, low-complexity adjunct for rapidly identifying high-risk patients during the initial ED evaluation. External multicenter validation is required before clinical adoption.

## Figures and Tables

**Table 1:** Baseline characteristics of the study population (N = 482).

Characteristic	Value
Demographics	
Age (years), mean ± SD	71.6 ± 15.2
Age (years), median (IQR)	75 (63–83)
Male, n (%)	248 (51.5)
Female, n (%)	234 (48.5)
Vital signs at presentation	
Systolic blood pressure (mmHg), mean ± SD	130.2 ± 28.2
Heart rate (/min), mean ± SD	88.5 ± 19.6
Laboratory findings	
Albumin (g/dL), mean ± SD	4.0 ± 0.6
PT-INR, mean ± SD	1.1 ± 0.2
WBC (×10^3^/µL), mean ± SD	10.6 ± 5.0
Total bilirubin (mg/dL), median (IQR)	2.2 (1.1–4.6)
Creatinine (mg/dL), median (IQR)	0.9 (0.7–1.1)
BUN (mg/dL), mean ± SD	18.3 ± 13.4
CRP (mg/dL), mean ± SD	5.6 ± 6.4
PLS4 components met	
Albumin < 3.5 g/dL, n (%)	93 (19.3)
SBP ≤ 100 mmHg, n (%)	66 (13.7)
HR ≥ 100/min, n (%)	131 (27.2)
PT-INR ≥ 1.2, n (%)	116 (24.1)
TG2018 severity grade	
Grade I/II/III, n (%)	254 (52.7)/120 (24.9)/108 (22.4)
Comorbidities	
Hypertension, n (%)	242 (50.2)
Diabetes mellitus, n (%)	125 (25.9)
Biliary drainage	
ERCP performed, n (%)	324 (67.2)
PTBD performed, n (%)	49 (10.2)
Outcomes	
ICU admission, n (%)	81 (16.8)
Vasopressor use, n (%)	53 (11.0)
Prolonged length of stay (≥14 days), n (%)	111 (23.0)
Intubation, n (%)	14 (2.9)
In-hospital mortality, n (%)	8 (1.7)
Mean hospital stay (days), mean ± SD	11.1 ± 12.0

Data are presented as mean ± standard deviation (SD), median (interquartile range, IQR), or n (%). BUN, blood urea nitrogen; CRP, C-reactive protein; ERCP, endoscopic retrograde cholangiopancreatography; PTBD, percutaneous transhepatic biliary drainage; ICU, intensive care unit. HR, heart rate; PT-INR, prothrombin time–international normalized ratio; SBP, systolic blood pressure; TG2018, Tokyo Guidelines 2018; WBC, white blood cell count.

**Table 2:** Univariable predictive performance (area under the ROC curve) of individual variables and Tokyo Guidelines 2018 severity grading for ICU admission and vasopressor use, with bootstrap 95% confidence intervals.

Variable	AUC—ICU Admission (95% CI)	AUC—Vasopressor Use (95% CI)
TG2018 severity grading	0.850 (0.801–0.896)	0.936 (0.920–0.951)
Albumin (g/dL)	0.771 (0.713–0.824)	0.820 (0.762–0.876)
PT-INR	0.754 (0.687–0.819)	0.796 (0.725–0.861)
Systolic blood pressure (mmHg)	0.737 (0.667–0.807)	0.807 (0.730–0.876)
Blood urea nitrogen (mg/dL)	0.713 (0.652–0.771)	0.771 (0.703–0.833)
Heart rate (/min)	0.650 (0.579–0.719)	0.712 (0.639–0.781)

AUC, area under the receiver-operating characteristic curve; TG2018, Tokyo Guidelines 2018; PT-INR, prothrombin time–international normalized ratio; ICU, intensive care unit. CI, confidence interval.

**Table 3:** Distribution of PLS4 score and event rates by score category (0–4 points; N = 482), with in-hospital mortality.

PLS4 Score	n (%)	ICU Admission (%)	Vasopressor Use (%)	Prolonged Stay ≥ 14 d (%)	Intubation, % (95% CI)	In-Hospital Mortality, n/N (%)
0	235 (48.8)	5.96	2.55	13.62	0.85 (0.23–3.05)	0/235 (0.0)
1	141 (29.3)	10.64	6.38	24.11	2.84 (1.11–7.07)	0/141 (0.0)
2	63 (13.1)	28.57	12.70	34.92	0.00 (0.00–5.75)	2/63 (3.2)
3	33 (6.8)	72.73	63.64	51.52	21.21 (10.68–37.75)	5/32 (15.6)
4	10 (2.1)	100.00	90.00	60.00	10.00 (1.79–40.42)	1/10 (10.0)

PLS4 assigns one point each for albumin < 3.5 g/dL, systolic blood pressure ≤ 100 mmHg, heart rate ≥ 100/min, and PT-INR ≥ 1.2 (range 0–4). Percentages represent within-score-category event rates; intubation rates are shown with Wilson score 95% confidence intervals.

**Table 4:** Comparison of area under the ROC curve (AUC) between PLS4 and Tokyo Guidelines 2018 (TG2018) severity grading for primary and exploratory outcomes, with bootstrap 95% confidence intervals, paired comparisons, and optimism-corrected estimates.

Outcome	TG2018 AUC (95% CI)	PLS4 AUC (95% CI)	ΔAUC	*p*	TG2018 opt.-corr.	PLS4 opt.-corr.
ICU admission (primary)	0.850 (0.801–0.896)	0.800 (0.736–0.860)	0.051	0.116	—	0.779
Vasopressor use	0.936 (0.920–0.951)	0.843 (0.770–0.904)	0.093	0.001	—	0.828
Prolonged length of stay (≥14 d)	0.680 (0.625–0.735)	0.669 (0.608–0.727)	0.011	0.717	—	0.664
Intubation (exploratory)	0.854 (0.749–0.912)	0.767 (0.616–0.897)	0.087	0.269	—	0.756

AUC, area under the receiver-operating characteristic curve; CI, confidence interval; opt.-corr., optimism-corrected; TG2018, Tokyo Guidelines 2018.

**Table 5:** Operating characteristics (sensitivity, specificity, positive predictive value, negative predictive value) of TG2018 and PLS4 at clinically relevant cut-off thresholds, with Wilson score 95% confidence intervals.

Outcome	Cut-off	Sensitivity (95% CI)	Specificity (95% CI)	PPV (95% CI)	NPV (95% CI)
ICU admission	TG2018 ≥ 2	0.901 (0.817–0.949)	0.613 (0.565–0.660)	0.320 (0.263–0.383)	0.969 (0.939–0.984)
	TG2018 = 3	0.753 (0.649–0.834)	0.883 (0.848–0.911)	0.565 (0.471–0.655)	0.947 (0.919–0.965)
	PLS4 ≥ 2	0.642 (0.533–0.738)	0.865 (0.828–0.895)	0.491 (0.397–0.584)	0.923 (0.891–0.946)
	PLS4 ≥ 3	0.420 (0.318–0.528)	0.978 (0.958–0.988)	0.791 (0.648–0.886)	0.893 (0.861–0.919)
Vasopressor use	TG2018 ≥ 2	1.000 (0.932–1.000)	0.592 (0.545–0.638)	0.232 (0.182–0.291)	1.000 (0.985–1.000)
	TG2018 = 3	1.000 (0.932–1.000)	0.872 (0.837–0.900)	0.491 (0.398–0.584)	1.000 (0.990–1.000)
	PLS4 ≥ 2	0.717 (0.584–0.820)	0.841 (0.804–0.873)	0.358 (0.274–0.453)	0.960 (0.935–0.976)
	PLS4 ≥ 3	0.566 (0.433–0.690)	0.970 (0.949–0.982)	0.698 (0.549–0.814)	0.948 (0.923–0.965)

CI, confidence interval; PPV, positive predictive value; NPV, negative predictive value; TG2018, Tokyo Guidelines 2018; ICU, intensive care unit.

**Table 6:** Timing of biliary decompression by TG2018 severity grade.

Procedure/Timing	Grade I	Grade II	Grade III
ERCP, n	176	84	64
Time to ERCP (h), median (IQR)	29.4 (19.2–54.1)	28.5 (19.1–69.7)	50.1 (25.2–115.8)
ERCP < 24 h, n (%)	66 (37.5)	30 (35.7)	16 (25.0)
ERCP 24–48 h, n (%)	56 (31.8)	25 (29.8)	14 (21.9)
ERCP > 48 h, n (%)	54 (30.7)	29 (34.5)	34 (53.1)
PTBD, n	17	9	23
Time to PTBD (h), median (IQR)	38.4 (21.4–63.3)	25.4 (17.1–41.3)	21.3 (12.0–67.1)

ERCP, endoscopic retrograde cholangiopancreatography; IQR, interquartile range; PTBD, percutaneous transhepatic biliary drainage; TG2018, Tokyo Guidelines 2018.

## Data Availability

The data used to support the findings of this study are available from the corresponding author upon request.

## Supplementary Materials

The following supporting information can be downloaded at: https://www.mdpi.com/article/10.3390/jcm15207842/s1, Figure S1. Flow diagram of patient selection. Table S1. Discrimination of equally weighted PLS4 versus coefficient-weighted logistic models using the same predictors, with and without BUN. Table S2. STROBE statement—checklist of items that should be included in reports of cohort studies.

## References

[1] Sokal A., Sauvanet A., Fantin B., de Lastours V. (2019). Acute cholangitis: Diagnosis and management. J. Visc. Surg..

[2] An Z., Braseth A.L., Sahar N. (2021). Acute Cholangitis: Causes, Diagnosis, and Management. Gastroenterol. Clin. N. Am..

[3] Cozma M.-A., Găman M.-A., Srichawla B.S., Dhali A., Manan M.R., Nahian A., Marsool M.D.M., Suteja R.C., Kutikuppala L.V.S., Kipkorir V. (2024). Acute cholangitis: A state-of-the-art review. Ann. Med. Surg..

[4] Lavillegrand J.-R., Mercier-Des-Rochettes E., Baron E., Pène F., Contou D., Favory R., Préau S., Galbois A., Molliere C., Miailhe A.-F. (2021). Acute cholangitis in intensive care units: Clinical, biological, microbiological spectrum and risk factors for mortality: A multicenter study. Crit. Care.

[5] Lee J.G. (2009). Diagnosis and management of acute cholangitis. Nat. Rev. Gastroenterol. Hepatol..

[6] Wada K., Takada T., Kawarada Y., Nimura Y., Miura F., Yoshida M., Mayumi T., Strasberg S., Pitt H.A., Gadacz T.R. (2007). Diagnostic criteria and severity assessment of acute cholangitis: Tokyo Guidelines. J. Hepato-Biliary-Pancreat. Surg..

[7] Kiriyama S., Kozaka K., Takada T., Strasberg S.M., Pitt H.A., Gabata T., Hata J., Liau K.-H., Miura F., Horiguchi A. (2018). Tokyo Guidelines 2018: Diagnostic criteria and severity grading of acute cholangitis (with videos). J. Hepato-Biliary-Pancreat. Sci..

[8] Mayumi T., Okamoto K., Takada T., Strasberg S.M., Solomkin J.S., Schlossberg D., Pitt H.A., Yoshida M., Gomi H., Miura F. (2018). Tokyo Guidelines 2018: Management bundles for acute cholangitis and cholecystitis. J. Hepato-Biliary-Pancreat. Sci..

[9] Yokoe M., Takada T., Mayumi T., Yoshida M., Hasegawa H., Norimizu S., Hayashi K., Umemura S., Orito E. (2011). Accuracy of the Tokyo Guidelines for the diagnosis of acute cholangitis and cholecystitis taking into consideration the clinical practice pattern in Japan. J. Hepato-Biliary-Pancreat. Sci..

[10] Fujii Y., Ohuchida J., Chijiiwa K., Yano K., Imamura N., Nagano M., Hiyoshi M., Otani K., Kai M., Kondo K. (2012). Verification of Tokyo Guidelines for diagnosis and management of acute cholangitis. J. Hepato-Biliary-Pancreat. Sci..

[11] Hudgi A., Cartelle A.L., Ahmed A., Alkaddour A., Palacio C., Vega K.J., Yap J.E.L. (2022). Tokyo Guidelines (TG18) for Acute Cholangitis Provide Improved Specificity and Accuracy Compared to Fellow Assessment. Cureus.

[12] Sun G., Han L., Yang Y., Linghu E., Li W., Cai F., Kong J., Wang X., Meng J., Du H. (2014). Comparison of two editions of Tokyo guidelines for the management of acute cholangitis. J. Hepato-Biliary-Pancreat. Sci..

[13] Yoga V., Fauzi A., Maulahela H., Shatri H., Yunir E., Waleleng B.J., Makmun D., Renaldi K., Rumende C.M., Sukamto S. (2025). Performance of Tokyo Guidelines 2018 and Predictors of Mortality in Acute Cholangitis Patients in Indonesia. Acta Med. Indones..

[14] Farooq U., Gondal A.B., Franco D., Tarar Z.I., Mahmood S. (2023). Validation of Tokyo guidelines 2018 for safety and mortality benefit from urgent ERCP in acute cholangitis across different age groups. J. Hepato-Biliary-Pancreat. Sci..

[15] Jung H.-M., Paik J., Lee M., Kim Y.W., Kim T.-Y. (2024). Clinical Utility of the Tokyo Guidelines 2018 for Acute Cholangitis in the Emergency Department and Comparison with Novel Markers (Neutrophil-to-Lymphocyte and Blood Nitrogen Urea-to-Albumin Ratios). J. Clin. Med..

[16] Zhang H.-Y., Lu Z.-Q., Wang G.-X., Xie M.-R., Li C.-S. (2021). Presepsin as a biomarker for risk stratification for acute cholangitis in emergency department: A single-center study. World J. Clin. Cases.

[17] Umefune G., Kogure H., Hamada T., Isayama H., Ishigaki K., Takagi K., Akiyama D., Watanabe T., Takahara N., Mizuno S. (2017). Procalcitonin is a useful biomarker to predict severe acute cholangitis: A single-center prospective study. J. Gastroenterol..

[18] Acehan F., Camli H., Kalkan C., Tez M., Demir B.F., Altiparmak E., Ates I. (2024). Red cell distribution width-to-albumin ratio is a simple promising prognostic marker in acute cholangitis requiring biliary drainage. Hepatobiliary Pancreat. Dis. Int..

[19] Li D., Sun J., Qi C., Fu X., Gao F. (2024). Predicting severity of inpatient acute cholangitis: Combined neutrophil-to-lymphocyte ratio and prognostic nutritional index. BMC Gastroenterol..

[20] Liu J., Wang P., Ji J. (2025). Neutrophil Percentage-To-Albumin Ratio is Associated with 30-Day All-Cause Mortality in Septic Cholangitis Patients: A Cohort Study. Shock.

[21] Lu Z.-Q., Zhang H.-Y., Su C.-F., Xing Y.-Y., Wang G.-X., Li C.-S. (2022). Optimal timing of biliary drainage based on the severity of acute cholangitis: A single-center retrospective cohort study. World J. Gastroenterol..

[22] Aboelsoud M., Siddique O., Morales A., Seol Y., Al-Qadi M. (2018). Early biliary drainage is associated with favourable outcomes in critically-ill patients with acute cholangitis. Prz. Gastroenterol..

[23] Parikh M.P., Wadhwa V., Thota P.N., Lopez R., Sanaka M.R.M. (2018). Outcomes Associated With Timing of ERCP in Acute Cholangitis Secondary to Choledocholithiasis. J. Clin. Gastroenterol..

[24] Park N., Lee S.H., You M.S., Kim J.S., Huh G., Chun J.W., Cho I.R., Paik W.H., Ryu J.K., Kim Y.-T. (2021). Optimal timing of endoscopic retrograde cholangiopancreatography for acute cholangitis associated with distal malignant biliary obstruction. BMC Gastroenterol..

[25] Seo Y.-J., Hadaya J., Sareh S., Aguayo E., de Virgilio C.M., Benharash P. (2020). National trends and outcomes in timing of ERCP in patients with cholangitis. Surgery.

[26] Gigot J.F., Leese T., Dereme T., Coutinho J., Castaing D., Bismuth H. (1989). Acute cholangitis. Multivariate analysis of risk factors. Ann. Surg..

[27] Tsuyuguchi T., Sugiyama H., Sakai Y., Nishikawa T., Yokosuka O., Mayumi T., Kiriyama S., Yokoe M., Takada T. (2012). Prognostic factors of acute cholangitis in cases managed using the Tokyo Guidelines. J. Hepato-Biliary-Pancreat. Sci..

[28] Touzani S., El Bouazzaoui A., Bouyarmane F., Faraj K., Houari N., Boukatta B., Kanjaa N. (2021). Factors Associated with Mortality in Severe Acute Cholangitis in a Moroccan Intensive Care Unit: A Retrospective Analysis of 140 Cases. Gastroenterol. Res. Pr..

[29] Schneider J., Hapfelmeier A., Thöres S., Obermeier A., Schulz C., Pförringer D., Nennstiel S., Spinner C., Schmid R.M., Algül H. (2016). Mortality Risk for Acute Cholangitis (MAC): A risk prediction model for in-hospital mortality in patients with acute cholangitis. BMC Gastroenterol..

[30] Hocine K., Briant A.R., Chaigneau T., Kam W., Collet T., Parienti J.-J., Piquet M.A., Dupont B. (2025). Early prediction of mortality in acute cholangitis: Elaboration of a new simple prognostic score. Hepatobiliary Pancreat. Dis. Int..

[31] Huang S., Zhou Y., Liang Y., Ye S., Zhu A., Li J., Bai X., Yue C., Feng Y. (2024). Machine-learning-derived online prediction models of outcomes for patients with cholelithiasis-induced acute cholangitis: Development and validation in two retrospective cohorts. EClinicalMedicine.

[32] Wolff R.F., Moons K.G., Riley R., Whiting P.F., Westwood M., Collins G.S., Reitsma J.B., Kleijnen J., Mallett S., PROBAST Group (2019). PROBAST: A Tool to Assess the Risk of Bias and Applicability of Prediction Model Studies. Ann. Intern. Med..

[33] Collins G.S., Reitsma J.B., Altman D.G., Moons K.G. (2015). Transparent reporting of a multivariable prediction model for individual prognosis or diagnosis (TRIPOD): The TRIPOD statement. BMJ.

[34] Riley R.D., Ensor J., I E Snell K., E Harrell F., Martin G.P., Reitsma J.B., Moons K.G.M., Collins G., van Smeden M. (2020). Calculating the sample size required for developing a clinical prediction model. BMJ.

[35] von Elm E., Altman D.G., Egger M., Pocock S.J., Gøtzsche P.C., Vandenbroucke J.P., STROBE Initiative (2008). The Strengthening the Reporting of Observational Studies in Epidemiology (STROBE) statement: Guidelines for reporting observational studies. J. Clin. Epidemiol..

[36] Groot O.Q., Bindels B.J.J., Ogink P.T., Kapoor N.D., Twining P.K., Collins A.K., Bongers M.E.R., Lans A., Oosterhoff J.H.F., Karhade A.V. (2021). Availability and reporting quality of external validations of machine-learning prediction models with orthopedic surgical outcomes: A systematic review. Acta Orthop..

[37] Priestap F., Kao R., Martin C.M. (2020). External validation of a prognostic model for intensive care unit mortality: A retrospective study using the Ontario Critical Care Information System. Can. J. Anaesth..

