# Supplementary material for: A Simplified Physiology–Laboratory Score (PLS4) for Early Risk Stratification in Acute Cholangitis
Source: J Clin Med. 2026 Oct 11;15(20):7842. doi: 10.3390/jcm15207842 (PMC13705153; doi:10.3390/jcm15207842)
Supplement: Supplementary file 1 [file jcm-15-07842-s001.zip › jcm-4550711-supplementary.pdf]

Supplementary Materials

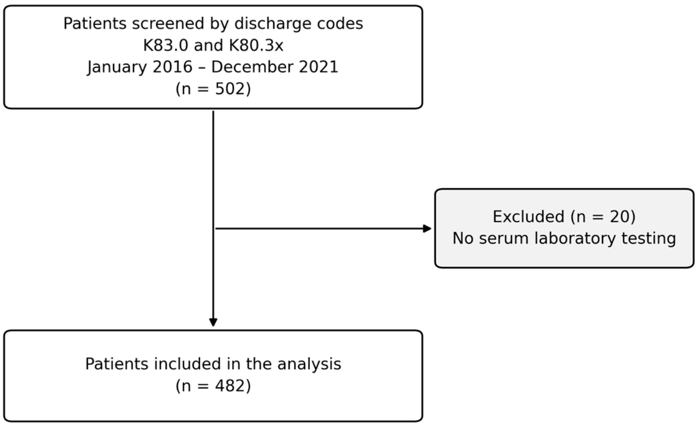

Figure S1. Flow diagram of patient selection. Patients were screened using discharge codes for acute cholangitis (K83.0) and choledocholithiasis with cholangitis (K80.3x) between January 2016 and December 2021; 20 patients without serum laboratory testing were excluded, leaving 482 patients for analysis.

Table S1. Discrimination of equally weighted PLS4 versus coefficient-weighted logistic models using the same predictors, with and without BUN.

Model	ICU admission AUC (95% CI)	ICU admission optimism-corrected	Vasopressor use AUC (95% CI)	Vasopressor use optimism-corrected
PLS4, equal weights (0–4 points)	0.800 (0.740–0.857)	0.779	0.843 (0.775–0.904)	0.828
Logistic model: albumin, SBP, HR, PT-INR (continuous)	0.808 (0.747–0.868)	0.801	0.889 (0.839–0.932)	0.882
Logistic model: albumin, SBP, HR, PT-INR, BUN (continuous)	0.820 (0.764–0.875)	0.811	0.891 (0.842–0.935)	0.883

AUCs with bootstrap 95% confidence intervals (2,000 outcome-stratified resamples). For the logistic models, optimism was estimated by refitting the model in 1,000 bootstrap samples and testing it in the original sample; for PLS4, the optimism-corrected AUC repeats variable and cut-off selection in each resample (main text, Table 4). Log-odds coefficients of the four-predictor model (per unit of albumin [g/dL], SBP [mmHg], HR [/min], and PT-INR) were -1.245, -0.0202, 0.0268, and 1.141 for ICU admission and -1.396, -0.0361, 0.0405, and 1.052 for vasopressor use. BUN, blood urea nitrogen; HR, heart rate; PT-INR, prothrombin time–international normalized ratio; SBP, systolic blood pressure.

Table S2. STROBE statement—checklist of items that should be included in reports of cohort studies.

Section/topic	Item	Recommendation	Reported in
Title and abstract	1	(a) Indicate the study's design with a commonly used term in the title or the abstract; (b) provide an informative and balanced summary of what was done and what was found	Abstract
Background/rationale	2	Explain the scientific background and rationale for the investigation being reported	Introduction

Objectives	3	State specific objectives, including any prespecified hypotheses	Introduction, last paragraph
Study design	4	Present key elements of study design early in the paper	Section 2.1
Setting	5	Describe the setting, locations, and relevant dates, including periods of recruitment, exposure, follow-up, and data collection	Section 2.1
Participants	6	(a) Give the eligibility criteria, and the sources and methods of selection of participants; describe methods of follow-up	Section 2.1; Figure S1
Variables	7	Clearly define all outcomes, exposures, predictors, potential confounders, and effect modifiers	Sections 2.2 and 2.3
Data sources/measurement	8	For each variable of interest, give sources of data and details of methods of assessment	Sections 2.1–2.3
Bias	9	Describe any efforts to address potential sources of bias	Sections 2.1 and 2.4; Section 4.1
Study size	10	Explain how the study size was arrived at	Sections 2.1 and 2.3
Quantitative variables	11	Explain how quantitative variables were handled in the analyses	Section 2.3
Statistical methods	12	(a) Describe all statistical methods; (b) methods used to examine subgroups and interactions; (c) how missing data were addressed; (d) how loss to follow-up was addressed; (e) sensitivity analyses	Section 2.4
Participants	13	(a) Report numbers of individuals at each stage of the study; (b) give reasons for non-participation at each stage; (c) consider use of a flow diagram	Sections 2.1 and 3.1; Figure S1
Descriptive data	14	(a) Give characteristics of study participants; (b) indicate the number of participants with missing data for each variable of interest	Table 1; Table 3 note
Outcome data	15	Report numbers of outcome events or summary measures over time	Sections 2.2, 3.1, and 3.5; Tables 1 and 3
Main results	16	(a) Give unadjusted estimates and, if applicable, adjusted estimates and their precision (e.g., 95% confidence interval); (b) report category boundaries when continuous variables were categorized; (c) if relevant, translate estimates of relative risk into absolute risk	Sections 3.2–3.4; Tables 2–4; Figure 1
Other analyses	17	Report other analyses done, e.g., analyses of subgroups and interactions, and sensitivity analyses	Sections 3.6–3.9; Tables 5 and 6; Figures 4 and 5; Table S1
Key results	18	Summarize key results with reference to study objectives	Section 4, first paragraph
Limitations	19	Discuss limitations of the study, taking into account sources of potential bias or imprecision	Section 4.1
Interpretation	20	Give a cautious overall interpretation of results considering objectives, limitations, multiplicity of analyses, results from similar studies, and other relevant evidence	Section 4
Generalizability	21	Discuss the generalizability (external validity) of the study results	Sections 4 and 4.1
Funding	22	Give the source of funding and the role of the funders for the present study	Funding statement

Adapted from von Elm E, et al. The Strengthening the Reporting of Observational Studies in Epidemiology (STROBE) statement: guidelines for reporting observational studies.
